# Molecular cloning, expression, and functional characterization of the β-agarase AgaB-4 from *Paenibacillus agarexedens*

**DOI:** 10.1186/s13568-018-0581-8

**Published:** 2018-03-28

**Authors:** Zeng-Weng Chen, Hui-Jie Lin, Wen-Cheng Huang, Shih-Ling Hsuan, Jiunn-Horng Lin, Jyh-Perng Wang

**Affiliations:** 1 Animal Technology Laboratories, Agricultural Technology Research Institute, No.52, Kedong 2nd Rd., Zhunan Township, Miaoli County 350, Taiwan, ROC; 2 Graduate Institute of Veterinary Pathobiology, National Chung Hsing University, 250 Kuo Kuang Road, Taichung 402, Taiwan, ROC

**Keywords:** β-Agarase, *Paenibacillus agarexedens*, Next-generation sequencing, Neoagarotetraose

## Abstract

In this study, a β-agarase gene, *agaB*-*4*, was isolated for the first time from the agar-degrading bacterium *Paenibacillus agarexedens* BCRC 17346 by using next-generation sequencing. *agaB*-*4* consists of 2652 bp and encodes an 883-amino acid protein with an 18-amino acid signal peptide. *agaB*-*4* without the signal peptide DNA was cloned and expressed in *Escherichia coli* BL21(DE3). His-tagged recombinant AgaB-4 (rAgaB-4) was purified from the soluble fraction of *E. coli* cell lysate through immobilized metal ion affinity chromatography. The optimal temperature and pH of rAgaB-4 were 55 °C and 6.0, respectively. The results of a substrate specificity test showed that rAgaB-4 could degrade agar, high-melting point agarose, and low-melting point agarose. The *V*_max_ and *K*_m_ of rAgaB-4 for low-melting point agarose were 183.45 U/mg and 3.60 mg/mL versus 874.61 U/mg and 9.29 mg/mL for high-melting point agarose, respectively. The main products of agar and agarose hydrolysis by rAgaB-4 were confirmed to be neoagarotetraose. Purified rAgaB-4 can be used in the recovery of DNA from agarose gels and has potential application in agar degradation for the production of neoagarotetraose.

**Electronic supplementary material:**

The online version of this article (10.1186/s13568-018-0581-8) contains supplementary material, which is available to authorized users.

## Introduction

Agar is a hydrophilic colloid extracted from the cell walls of red algae (Rhodophyceae), such as *Gelidium* spp., *Gracilaria* spp., and *Porphyra* spp. It is a heterogeneous polysaccharide which consists of agarose and porphyran (Chi et al. 2012). Agarose is a neutral polysaccharide that forms a gel; its molecular weight is approximately 120 kDa; and it consists of alternating β-d-galactose and 3,6-anhydro-α-l-galactopyranose linked by α-1,3 and β-1,4 glycosidic bonds (Armisén 1991; Yun et al. 2017). Porphyran, the non-gelling fraction, is a linear sulfated galactan; its composition is similar to that of agarose, except that some 3,6-anhydro-α-l-galactose are replaced with α-L-galactose-6-sulfate (Knutsen et al. 1994; Chi et al. 2012).

Agarases are enzymes that catalyze the hydrolysis of agar into oligosaccharides; these enzymes cleave glycosidic bonds at different positions. Thus, agarases can be classified into α-agarases (EC 3.2.1.158), β-agarases (EC 3.2.1.81), and β-porphyranases (EC 3.2.1.178) according to the cleavage pattern (Chi et al. 2012). α-Agarases act on the α-1,3 glycosidic bonds of agarose, producing agaro-oligosaccharides with a 3,6-anhydro-α-l-galactose residue at the reducing end. β-agarases, on the other hand, act on the β-1,4 glycosidic bonds of agarose, producing neoagaro-oligosaccharides with a d-galactose residue at the reducing end (Fu and Kim 2010). β-porphyranases act on the β-1,4 glycosidic bonds of porphyran, producing oligosaccharides with a d-galactose residue at the reducing end.

Various microbes from seawater, marine sediments, seaweed, marine mollusks, soil, solar salt, city drain water, and hot spring produce agarases. Seawater isolates, including *Alteromonas agarlyticus* GJ1B (Potin et al. 1993) and *Thalassomonas* sp. JAMB-A33 (Ohta et al. 2005), produce α-agarases. Based on the amino acid sequence similarity, known α-agarases belong to the glycoside hydrolase (GH) family GH96. Compared with the source of α-agarases, more bacterial strains produce β-agarases, such as *Vibrio* sp. JT0107 (Sugano et al. 1993) and *Catenovulum* sp. X3 (Xie et al. 2013) from seawater; *Vibrio* sp. PO-303 (Dong et al. 2007) and *Agarivorans* sp. HZ105 (Hu et al. 2009) from marine sediments; *Vibrio* sp. AP-2 (Aoki et al. 1990) and *Pseudoalteromonas antarctica* N-1 (Vera et al. 1998) from seaweed; *Agarivorans albus* YKW-34 (Fu et al. 2008) from marine mollusks; *Paenibacillus* sp. SSG-1 (Song et al. 2014) and *Alteromonas* sp. E-1 (Kirimura et al. 1999) from soil; *Halococcus* sp. 197A (Minegishi et al. 2013) from solar salt; *Alcaligenes* sp. Yen (Sie et al. 2009) from city drain water; and *Bacillus* sp. BI-3 (Li et al. 2014) from hot spring. Based on the amino acid sequence similarity, known β-agarases are classified into the four GH families of GH16, GH50, GH86, and GH118 (Lombard et al. 2014). Unlike the source of β-agarases, only two bacterial strains, including *Zobellia galactanivorans* DSM 12802 from the red alga and *Bacteroides plebeius* DSM 17135 from Japanese individuals (Hehemann et al. 2010, 2012), produce β-porphyranases. Based on the amino acid sequence similarity, known β-porphyranases belong to the GH families of GH16 and GH86.

Previous studies reported that α-agarases and β-agarases have various applications, for example, those in the recovery of DNA from agarose gel (Finkelstein and Rownd 1978), preparation of seaweed protoplasts (Araki et al. 1998), and production of agar-derived oligosaccharides (Fu and Kim 2010). Studies have shown that the oligosaccharides generated by the hydrolysis of agar or seaweed polysaccharide crude extracts by agarases have numerous biological activities, such as antioxidative activity (Wu and Pan 2004), hepatoprotective potential (Chen et al. 2006), immunostimulatory activity (Lee et al. 2017), antiobesity effect (Hong et al. 2017a), whitening effect on melanoma cells (Jang et al. 2009), moisturizing effect on skin (Kobayashi et al. 1997), and prebiotic effect (Hu et al. 2006). Hence, agar-derived oligosaccharides can be used as new-generation, high-value functional oligosaccharides in cosmetic, health food, and pharmaceutical industries.

In the present study, the newly identified β-agarase gene *agaB*-*4* from *Paenibacillus agarexedens* BCRC 17346 was cloned and expressed in the cytoplasm of *Escherichia coli* BL21(DE3). The characteristics and potential applications of the purified recombinant enzyme were also analyzed.

## Materials and methods

### Bacterial strains, plasmids, and culture condition

*Paenibacillus agarexedens* BCRC 17346 was purchased from BCRC (Bioresource Collection and Research Center, Hsinchu, Taiwan) and was used for the isolation of genomic DNA. *Escherichia coli* ECOS™ 9-5 (Yeastern, Taipei, Taiwan) was used for the propagation and manipulation of recombinant DNA. *E. coli* BL21(DE3) (Merck Millipore, Darmstadt, Germany) was used as the expression host. pJET1.2 (Fermentas, Maryland, USA) and pET-29a(+) (Merck Millipore) were used as cloning and expression vectors, respectively. *P. agarexedens* BCRC 17346 was cultured at 30 °C in nutrient broth medium supplemented with 0.1% (w/v) urea and 1% (w/v) glucose. *E. coli* was grown at 37 °C in Luria–Bertani (LB) medium (Difico, Detroit, USA) containing 30 μg/mL kanamycin, when required.

### General DNA techniques

Bacterial genomic DNA was isolated using the DNeasy Blood & Tissue Kit (Qiagen, Hilden, Germany). Plasmid DNA was isolated using the Plasmid Miniprep Purification Kit II (GMbiolab, Taichung, Taiwan) according to the manufacturer’s instructions. DNA fragments were amplified using GDP-HiFi DNA Polymerase (Genedirex, Las Vegas, USA) according to the manufacturer’s recommendations. All polymerase chain reactions (PCRs) were performed on a TProfessional TRIO thermocycler (Biometra GmbH, Göttingen, Germany). PCR products were purified using a PCR Clean-Up Kit (GMbiolab). Restriction enzyme digestions were performed according to the supplier’s recommendations (Thermo Fisher Scientific, Waltham, USA). DNA fragments were recovered from gels by using a Gel Elution Kit (GMbiolab). DNA ligation reactions were performed using a DNA Ligation Kit (Yeastern). The purified PCR product was cloned into pJET1.2 by using a CloneJET PCR Cloning Kit (Fermentas) according to the manufacturer’s recommendations. Plasmids were introduced into *E. coli* through heat shock transformation according to the manufacturer’s instructions.

### Whole-genome sequencing of *P. agarexedens*

The genomic DNA of *P. agarexedens* was isolated and quantified using a Quant-iT dsDNA BR assay (Thermo Fisher Scientific). The quality of the extracted genomic DNA was verified on a 0.6% agarose gel. DNA libraries were constructed using an Illumina TruSeq DNA LT Sample Prep Kit. Mate-pair libraries were constructed using an Illumina Mate Pair Library Prep Kit v2. For the de novo assembly of the complete genome, we used Velvet (Zerbino and Birney 2008) to assemble paired-end and mate-pair reads to scaffolds. Genes on these assembled scaffolds were predicted using GeneMark.hmm (Besemer and Borodovsky 1999) and were annotated using a BLAST (blastp) search against the NCBI nr protein database, with an e-value cutoff of 0.00001. For the functional annotation of predicted genes, the accession numbers from BLAST hits were mapped to GO terms by querying the GO database (Ashburner et al. 2000). Enzyme code annotations were retrieved by mapping to GO terms, and enzyme codes were retrieved by querying the GO database.

### Construction of expression vector

Based on the results of whole-genome sequencing of *P. agarexedens*, the forward primer PBAGA4F (5′-GATATAGGTACCGCCACGCCGTTCCCTACTC-3′, *Kpn*I site underlined) and the reverse primer PBAGA4R (5′-CAATATCTCGAG*TTA***GTGGTGGTGGTGGTGGTG**CTTTGAGATTAGCAGACGATCCATTA-3′; *Xho*I site underlined, stop codon in italics, and His tag DNA in bold) were designed and used to amplify the 2598-bp DNA fragment encoding the mature β-agarase AgaB-4 lacking the predicted signal peptide through PCR. This fragment was generated from the genomic DNA of *P. agarexedens* BCRC 17346. The PCR product was purified and then ligated to pJET1.2. The ligation mixture was transformed into *E. coli* ECOS™ 9-5 competent cells for the generation of recombinant plasmids. The recombinant plasmids were confirmed by DNA sequencing. The resulting plasmid containing the agarase DNA fragment was named pJET-AGAB-4. The agarase DNA fragment was excised from pJET1.2 by using *Kpn*I and *Xho*I and was subsequently subcloned into pET-29a(+) at the corresponding restriction sites. The recombinant plasmid, designated pET-AgaB-4, was confirmed by DNA sequencing and was then transformed into *E. coli* BL21(DE3).

### Expression and detection of rAgaB-4 in the soluble fraction

*E. coli* BL21(DE3)(pET-AgaB-4) cells were cultured in LB medium containing kanamycin (30 μg/mL), with shaking at 37 °C. On the next day, 0.1 mL of the overnight culture was inoculated into 10 mL of LB medium containing kanamycin (30 μg/mL) and was grown at various temperatures (37, 30, 25, 20, 16 °C), with shaking. When the optical density at 600 nm (OD_600_) of the cultures reached 0.4–0.6, isopropyl-β-d-thiogalactopyranoside (IPTG) at a final concentration of 0.1 mM was added. After 4 and 24-h incubation, cells were harvested by centrifugation at 10,000×*g* for 10 min at 4 °C and were then resuspended in lysis buffer (50 mM Tris–HCl and 500 mM NaCl, pH 8.0). Cells were lysed by sonication in an ice water bath. The suspensions (total cell lysate) were centrifuged at 10,000×*g* for 10 min at 4 °C. The clear supernatant (soluble fraction) was collected, and the remaining pellet (insoluble fraction) was resuspended in an equal volume of lysis buffer. Equal volumes of the total cell lysate, soluble fraction, and insoluble fraction were analyzed through 12.5% sodium dodecyl sulfate polyacrylamide gel electrophoresis (SDS-PAGE) (Laemmli 1970), using a minigel apparatus (model AE-6450; ATTO, Tokyo, Japan).

### Western blot analysis

Protein samples were separated through 12.5% SDS-PAGE. After electrophoresis, proteins were electrophoretically transferred onto methanol-activated polyvinylidene fluoride membrane (Merck Millipore). The membrane was blocked with 5% skim milk in phosphate-buffered saline and incubated with the Penta·His antibody (1:20,000) (Qiagen), followed by incubation with an alkaline phosphatase-conjugated anti-mouse antibody (1:20,000) (Bethyl, Montgomery, USA). Immunoreactive bands were visualized using BCIP/NBT substrate solution (PerkinElmer, Waltham, USA).

### Purification of rAgaB-4

*E. coli* BL21(DE3)(pET-AgaB-4) cells were cultured in 1 L of LB broth containing kanamycin (30 μg/mL), with shaking at 37 °C. Cells were cultured to an OD_600_ of 0.4–0.6. Subsequently, IPTG (0.1 mM) was added to induce rAgaB-4 expression at 20 °C for 24 h. Cells were harvested by centrifugation at 8000×*g* for 30 min and were then resuspended in lysis buffer. The cell suspension was disrupted using Constant Cell Disruption Systems (Constant Systems Ltd, Warwick, UK). The cell lysate was centrifuged at 8000×*g* for 15 min at 4 °C, and the resulting supernatant was filtered through a 0.22-μm membrane and applied to a 5-mL HiTrap™ excel affinity chromatography column (GE Healthcare, Uppsala, Sweden) according to the manufacture’s instruction. The purity of the eluted fusion protein was analyzed through 12.5% SDS-PAGE, and the protein concentration was determined using a Protein Quantification Assay Kit (MACHEREY–NAGEL, Düren, Germany).

### Enzyme activity measurements

Agarase activity was measured by determining the amount of reducing sugars generated from hydrolysis, according to the DNS method developed by Miller (1959), with minor modifications. Briefly, 50 μL of suitably diluted rAgaB-4 solution was mixed with 950 μL of phosphate buffer (50 mM, pH 6) containing 0.2% (w/v) low-melting point (LMP) agarose. After incubation at 40 °C for 10 min, the sample was mixed with 1.0 mL of 3,5-dinitrosalicylic acid reagent solution, heated in a boiling water bath for 10 min, and then cooled in an ice water bath. Absorbance (OD) readings at 540 nm were obtained on an Infinite 200 PRO microplate reader (Tecan Group Ltd, Männedorf, Switzerland). The amount of enzyme required to produce 1 μmol d-galactose per min under the assay conditions was defined as one unit (U) of agarase. d-galactose was used as a reference reducing sugar for preparing the standard curve.

### Effects of pH and temperature on agarase activity and stability

The effect of pH on rAgaB-4 activity was assayed at 40 °C in 50 mM buffer solutions containing 0.2% LMP agarose and 1.51 μg of purified rAgaB-4 with a pH range of 3–10 (at 1.0 intervals). The buffer solutions used were citric acid/sodium citrate buffer (pH 3–6), phosphate buffer (pH 6–8), and glycine–NaOH buffer (pH 9–10). The effect of temperature on rAgaB-4 activity was determined by monitoring agarase activity at temperatures ranging from 20 to 80 °C in 50 mM phosphate buffer (pH 6) containing 0.2% LMP agarose and 1.51 μg of purified rAgaB-4 for 10 min. The thermostability of rAgaB-4 was determined by measuring the residual enzyme activity after incubation at temperatures ranging from 20 to 80 °C in 50 mM phosphate buffer (pH 6) containing 0.2% LMP agarose and 1.51 μg of purified rAgaB-4 for 1 h.

### Effect of various metal ions and ethylenediaminetetraacetic acid (EDTA) on enzyme activity

The effects of various metal ions and EDTA on rAgaB-4 activity were assayed in 50 mM sodium phosphate buffer (pH 6) containing 0.2% LMP agarose and 1.91 μg of purified rAgaB-4 by adding metal ions or EDTA at a final concentration of 1 mM. Hydrolysis reactions were performed at 55 °C for 10 min. Relative activity was calculated as the enzyme activity of rAgaB-4 with added metal ion or EDTA/activity of rAgaB-4 × 100.

### Substrate specificity of rAgaB-4

The substrate specificity of rAgaB-4 was measured using high-melting point (HMP) agarose, LMP agarose, agar, sodium alginate, carrageenan, soluble starch, and sodium carboxymethyl cellulose. Hydrolysis reactions were performed at 55 °C for 10 min in 50 mM sodium phosphate buffer (pH 6) containing 0.2% substrates and 1.91 μg of purified rAgaB-4. Relative activity was defied as the percentage of activity determined with the respect to the maximum agarase activity.

### Determination of kinetic parameters

The kinetic parameters of purified rAgaB-4 (1.54 μg) were determined in 50 mM phosphate buffer (pH 6) containing LMP agarose and HMP agarose (molecular mass, 120 kDa), ranging in concentration from 2 to 30 mg/mL. The reaction mixture was incubated at 55 °C for 10 min. *K*_m_ and *V*_max_ for LMP agarose and HMP agarose were determined from Lineweaver–Burk plots using SigmaPlot 12 software (Systat Software, San Jose, USA). Subsequently, the *K*_cat_ (turnover number) and *K*_cat_/*K*_m_ (catalytic efficiency) values were calculated based on the *V*_max_, *K*_m_, and [E] (concentration of rAgaB-4) values.

### Thin layer chromatography analysis of hydrolysis products

The products of LMP agarose, HMP agarose, and agar hydrolysis by rAgaB-4 were detected using thin layer chromatography (TLC) performed on silica gel 60 plates (Merck Millipore) as previously described (Li et al. 2014) with some modifications. Hydrolysis reactions were conducted at 40 °C for 24 h in 50 mM sodium phosphate buffer (pH 6) containing 1% (w/v) LMP agarose, HMP agarose and agar with 1.54 μg of purified rAgaB-4, respectively. The reaction mixtures were centrifuged at 20,630×*g* for 10 min at 4 °C to pellet the undigested agarose and agar. Subsequently, 2 μL of each supernatant was applied to a silica gel 60 plate and was developed using an *n*-butanol-acetic acid-water solution (2:2:1, by volume). The developed oligosaccharides were detected by spraying the plate with aniline phthalate solution (Merck Millipore) and by heating it on a hot plate at 180 °C.

### Evaluation of rAgaB-4 ability for DNA recovery from gel

The pUC19 plasmid (2.5 μg) was embedded in 1% LMP agarose. The agarose containing the pUC19 plasmid was incubated at 70 °C for 10 min and was treated with 1 U rAgaB-4 at 40 °C for 1 h. The mixture was centrifuged at 20,630×*g* for 10 min at 4 °C to remove the undigested residue. The DNA in the supernatant was precipitated by adding 0.6 volumes of isopropanol in the presence of 2.5 M ammonium acetate and 1 μg/μL glycogen. The mixture was centrifuged at 20,630×*g* for 10 min at 4 °C to pellet the DNA. The precipitated DNA was washed twice with 70% ethanol, dried, and dissolved in sterile Tris–HCl buffer (pH 8.0). Equal amounts of recovered DNA and the original pUC19 plasmid were analyzed through agarose gel electrophoresis.

### Nucleotide sequence accession number

The nucleotide sequence of *agaB*-*4* reported in this study has been submitted to the GenBank database under the accession number MF998080.

## Results

### In silico analysis and cloning of the β-agarase gene *agaB*-*4*

The bioinformatics analysis of whole-genome sequencing data revealed that *P. agarexedens* may have four β-agarase genes (data not shown). According to the number of nucleotides in each gene, the genes were named in the descending order of size as *agaB*-*1*, *agaB*-*2*, *agaB*-*3*, and *agaB*-*4.* The present study focused on *agaB*-*4.* According to the bioinformatics analysis, the total length of *agaB*-*4* is 2652 bp, and its start and stop codons are ATG and TAA, respectively; this gene encodes an 883-amino acid protein. Signal peptide prediction (SignalP 4.1 Server, http://www.cbs.dtu.dk/services/SignalP/) revealed that AgaB-4 may be a secretory protein with an N-terminal signal peptide (MILAIIAGLTGQPGAAAA) consisting of 18 amino acids, and the cleavage site of the signal peptidase is located between Ala^18^ and Ala^19^. The molecular weight of the mature protein without the signal peptide is 94,136 Da, and the predicted isoelectric point is 5.57.

Amino acid sequence similarities searches were performed using the BLASTP program in NCBI. The results showed that the sequence of AgaB-4 was the most similar to that of agarase AgaW (GenBank accession No. AKV62624) from the soil bacterium *Cohnella* sp. LGH (Li et al. 2015), which belongs to the GH50 family. The degree of identity and similarity between AgaB-4 and AgaW was 93.6 and 97.5%, respectively. Amino acid sequence alignment of AgaB-4 was performed with agarase AgaW, Aga50D (GenBank accession No. ABD81904) (Kim et al. 2010), AgWH50A (GenBank accession No. AFP32918) (Liu et al. 2014), and HZ2 (GenBank accession No. ADY17919) (Lin et al. 2012), which all belong to the GH50 family. The results revealed higher conservation of the C-terminal sequence in these GH50 family members (Additional file 1: Figure S1). According to the amino acid sequence alignment results, AgaB-4 was determined to belong to the GH50 family.

To investigate whether the protein encoded by *agaB*-*4* exhibits the enzymatic activity of β-agarase, we conducted gene cloning, expression, and purification of rAgaB-4; and subsequently analyzed the enzyme properties. For gene cloning, specific primers were designed and used to amplify the DNA fragment encoding the mature AgaB-4 through PCR. The PCR product was then cloned into pJET1.2 to yield pJET-AGAB-4. Subsequently, the agarase DNA fragment was excised from pJET-AGAB-4 and inserted into pET-29a(+) to yield the agarase expression vector pET-AGAB-4.

### Expression and purification of rAgaB-4

**Fig. 1:**
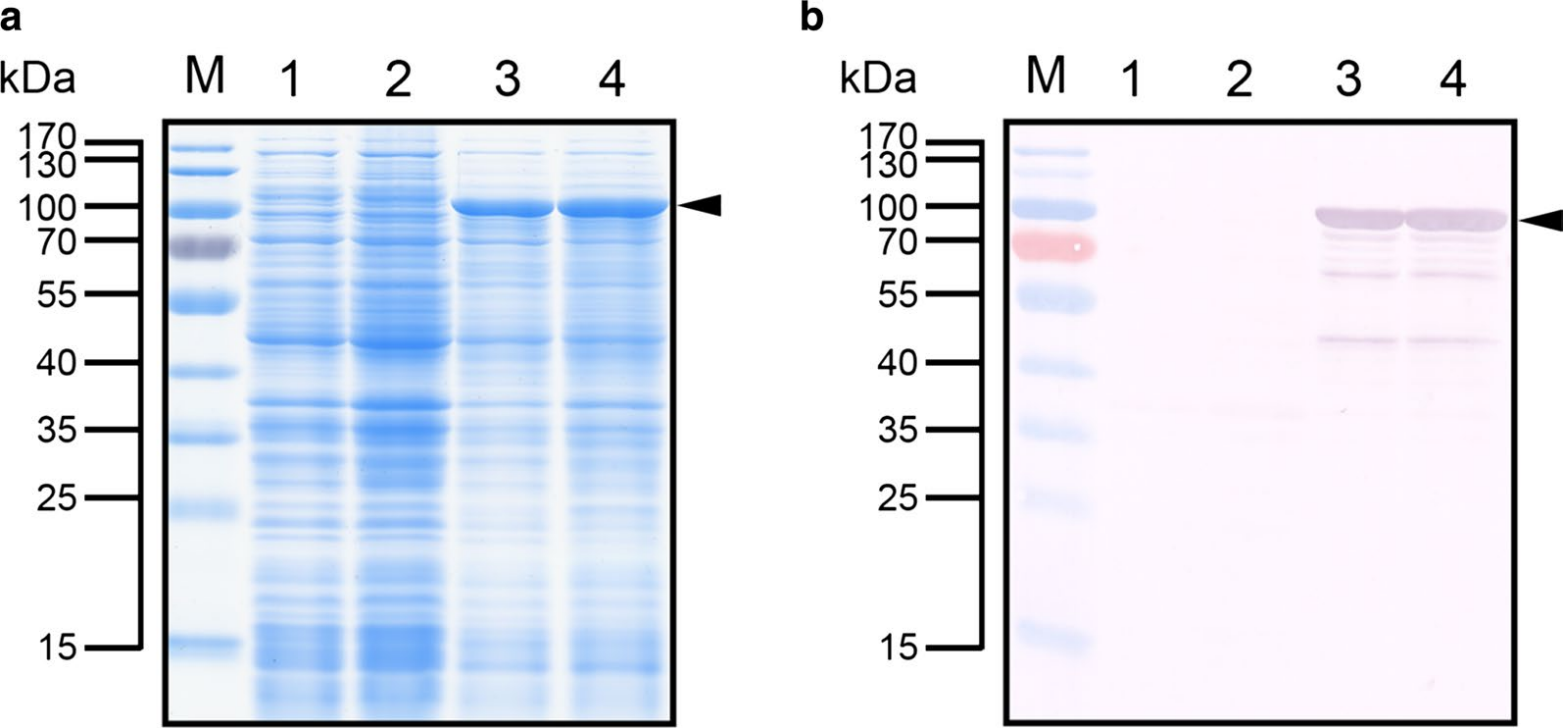
**a** SDS-PAGE and **b** Western blot analyses of rAgaB-4 expression in *E. coli* BL21(DE3). Lane M PageRuler™ Prestained Protein Ladder; lanes 1 and 2 total cell lysates of *E. coli* BL21(DE3) containing pET-29a(+) induced by IPTG at 37 °C for 4 and 24 h, respectively; lanes 3 and 4 total cell lysates of *E. coli* BL21(DE3) containing pET-AGAB-4 induced by IPTG at 37 °C for 4 and 24 h, respectively. Arrows indicate rAgaB-4 expression

**Fig. 2:**
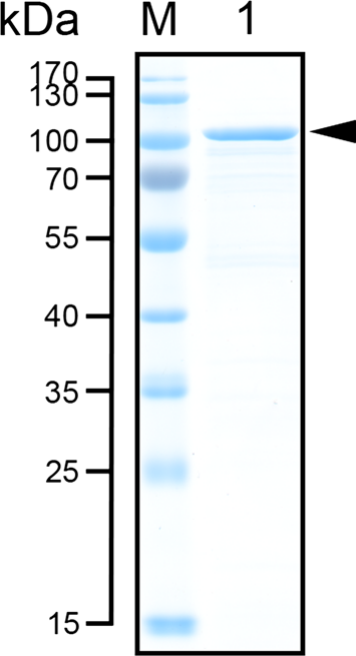
SDS-PAGE analysis of purified rAgaB-4. Lane M PageRuler™ Prestained Protein Ladder; lane 1 purified rAgaB-4

The agarase expression vector pET-AGAB-4 was transformed into *E. coli* BL21(DE3) cells, and IPTG was then added to induce protein expression. According to the results of SDS-PAGE and Western blot analyses, compared with *E. coli* BL21(DE3) transformants containing pET-29a(+), *E. coli* BL21(DE3) transformants containing the expression vector pET-AGAB-4 expressed the rAgaB-4 with the His-tag (attached to the C-terminal end); the molecular weight of the band was similar to the expected molecular weight of 97.32 kDa (Fig. 1). When protein expression was induced at 37 °C, rAgaB-4 mainly existed in the intracellular insoluble fraction. Protein expression induction at 20 °C and 16 °C significantly improved the solubility of rAgaB-4 (Additional file 1: Figure S2). This result suggests that lowering the induction temperature increases the solubility of rAgaB-4 expressed in *E. coli*. rAgaB-4 in intracellular soluble fraction from *E. coli* was purified using immobilized metal ion affinity chromatography. In SDS-PAGE, a single band with a molecular weight of approximately 100 kDa was observed, representing the homogenous composition of the purified protein (Fig. 2).

### Characteristics of rAgaB-4

**Fig. 3:**
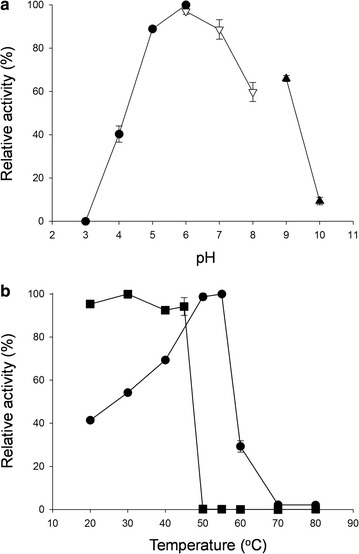
Effects of pH and temperature on rAgaB-4 activity. **a** Effects of pH on rAgaB-4 activity. The buffer solutions used for different pH were 50 mM citric acid/sodium citrate buffer (pH 3–6), phosphate buffer (pH 6–8), and glycine-NaOH buffer (pH 9–10). **b** Effects of temperature on rAgaB-4 activity and stability. Filled circle, optimal temperature; Filled square, thermostability. Error bars represent standard errors from triplicate experiments

**Fig. 4:**
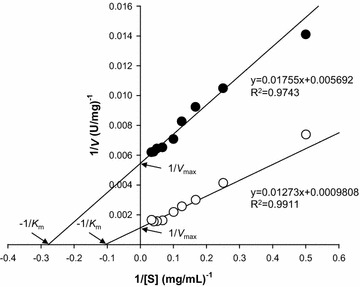
Lineweaver–Burk plots for determining the kinetic parameters of rAgaB-4 acting on low-melting point agarose (open circle) and high-melting point agarose (closed circle). *V* velocity; *S* substrate concentration. All data are mean values from triplicate experiments

**Fig. 5:**
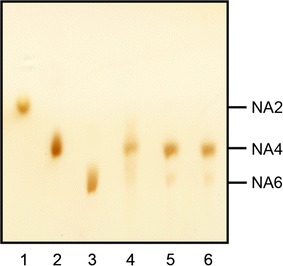
Thin layer chromatography analysis of the products of low-melting point agarose, high-melting point agarose, and agar hydrolysis by rAgaB-4. Lane 1 neoagarobiose (NA2); lane 2 neoagarotetraose (NA4); lane 3 neoagarohexaose (NA6); lane 4 hydrolysis products from low-melting point agarose; lane 5 hydrolysis products from high-melting point agarose; lane 6 hydrolysis products from agar

The optimal pH of rAgaB-4 was 6; more than 90% of enzyme activity could be maintained over a pH range of 5–7 (Fig. 3a). The optimal temperature of the enzyme was 55 °C; when the temperature increased to 60 °C, enzyme activity decreased sharply. When the temperature increased to up to 70 °C, enzyme activity was almost undetectable (Fig. 3b). The effect of temperature on enzyme stability was also investigated. The results indicated that when the enzyme was incubated at a temperature less than 45 °C for 1 h, 94% of enzyme activity was maintained. However, when the enzyme was incubated at a temperature higher than 50 °C for 1 h, the enzyme became completely inactivated (Fig. 3b). Moreover, the results showed that several metal ions, including Cu^2+^, K^+^, Fe^2+^, Ba^2+^, Na^+^, Sr^2+^, Co^2+^, Mg^2+^, Mn^2+^, Ca^2+^, and Al^3+^, may enhance the activity of rAgaB-4. Among these ions, Mn^2+^ was the most effective in enhancing enzyme activity, which increased up to 95% with Mn^2+^ addition. On the other hand, no significant activation or inhibition was observed by Zn^2+^ and EDTA (Table 1). The results of a substrate specificity test showed that the activity of rAgaB-4 was the highest for HMP agarose hydrolysis. Moreover, the relative enzyme activity for LMP agarose and agar was 62 and 94%, respectively. However; rAgaB-4 could not hydrolyze sodium alginate, carrageenan, soluble starch, and sodium carboxymethyl cellulose (Table 2). According to the Lineweaver–Burk plots, *V*_max_ and *K*_m_ of rAgaB-4 for LMP agarose were 183.45 U/mg and 3.60 mg/mL, respectively, while those for HMP agarose were 874.61 U/mg and 9.29 mg/mL, respectively (Fig. 4). The calculated *K*_cat_ and *K*_cat_/*K*_m_ values were, respectively 2.98 × 10^2^/s and 9.92 × 10^6^/s/M for LMP agarose, and 1.42 × 10^3^/s and 1.83 × 10^7^/s/M for HMP agarose. The results of kinetic analysis revealed that rAgaB-4 had higher catalytic efficiency (*K*_cat_/*K*_m_) toward HMP agarose than that toward LMP agarose. TLC analysis showed that neoagarotetraose was the main product formed from the hydrolysis of LMP agarose, HMP agarose and agar by rAgaB-4 (Fig. 5).

**Table 1:** Effects of various metal ions and EDTA on rAgaB-4 activity

Metal ion	Relative activity (%)^a^
None	100
Cu^2+^	113
K^+^	121
Zn^2+^	107
Fe^2+^	112
Ba^2+^	122
Na^+^	124
Sr^2+^	124
Co^2+^	140
Mg^2+^	116
Mn^2+^	195
Ca^2+^	121
Al^3+^	111
EDTA	105

^a^The enzymatic activity measured in the absence of metal ions or EDTA was defined as 100%. All data are mean values from triplicate experiments

**Table 2:** Substrate specificity of rAgaB-4

Substrate	Relative activity (%)^a^
High-melting point agarose	100
Low-melting point agarose	62
Agar	94
Sodium alginate	1
Carrageenan	0
Soluble starch (from potato)	0
Sodium carboxymethyl cellulose	1

^a^The highest enzymatic activity was defined as 100%. All data are mean values from triplicate experiments

### rAgaB-4 is used in the recovery of DNA from gels

The feasibility of using rAgaB-4 to recover DNA from gels was tested using pUC19 DNA as the model DNA. The results showed that rAgaB-4 could be used for the recovery of DNA from gels, and the recovery efficiency was more than 95% (Additional file 1: Figure S3).

## Discussion

*P. agarexedens* is an agar-degrading bacterium that was isolated from meadow soil by Miehlmanni in 1972 (Uetanabaro et al. 2003). The culture conditions, physiological characteristics, and genetic characteristics of this bacterium have been studied. Agarase production by this bacterium has been proven by the appearance of depressions around bacterial colonies when cultured in solid culture medium containing agar. However, the type of agarase produced by this bacterium and the cellular localization, gene and enzyme characteristics, and applications of agarase have yet to be analyzed. In this study, the agarase gene of this bacterium was explored through whole-genome sequencing and bioinformatics analysis. Subsequently, cloning and expression of *agaB*-*4* were performed to reveal the characteristics of the recombinant enzyme.

The sequence of AgaB-4 reported in this study was the most similar to that of agarase AgaW from *Cohnella* sp. LGH, which belongs to the GH50 family. In addition, the partial C-terminal sequence is conserved among various GH50 family members. Therefore, AgaB-4 was determined to belong to the GH50 family. To date, according to the CAZy database (http://www.cazy.org/Glycoside-Hydrolases.html), the GH50 family is composed of 476 types of β-agarases; the products of agarose hydrolysis by agarases belonging to the GH50 family are neoagarobiose, neoagarotetraose, or a mixture of the two compounds. The main hydrolysis products of AgaB-4 and AgaW from *Cohnella* sp. LGH are identical, namely neoagarotetraose. However, these enzymes still differ in terms of specific properties. For example, the optimal pH of AgaB-4 and AgaW is 6 and 7, respectively. When incubated at 50 °C for 1 h, AgaB-4 is completely inactivated. By contrast, AgaW can maintain 50% activity. This finding shows that the difference in amino acid sequence affects enzyme properties. Currently, of the agarases in the GH50 family, only the structure of Aga50D from *Saccharophagus degradans* has been studied (Pluvinage et al. 2013). Its possible catalytic residues are Glu-534 and Glu-695. Compared with these catalytic residues, the possible catalytic residues of AgaB-4 are Glu-455 and Glu-617, indicating a remarkable conservation of catalytic residues. Additional studies should evaluate the structure and function of AgaB-4 to understand its possible catalytic mechanism.

The production of agaro-oligosaccharides or neoagaro-oligosaccharides by using the enzymatic hydrolysis method has several advantages over the acid hydrolysis method (Chen et al. 2004, 2005; Yang et al. 2009). The advantages include (1) enzymes selectively cleave specific glycosidic bonds to produce oligosaccharides with a specific degree of polymerization; (2) the degradation conditions are easier to control; (3) the optimal temperature for enzyme activity is lower than the optimal temperature for acid hydrolysis, reducing energy consumption; (4) the operation process of the enzymatic hydrolysis method is simpler than that of the acid hydrolysis method, without the need for reactions between acid and alkali and desalination; (5) no acids are required for the enzymatic hydrolysis method; thus, this method is safer and less likely to pollute the environment; (6) and enzymatic hydrolysis can produce agaro-oligosaccharides or neoagaro-oligosaccharides (Fu and Kim 2010). Acid hydrolysis can only produce agaro-oligosaccharides and cannot produce products with a uniform degree of polymerization; the polymerization degree is approximately 2–22 (Chen et al. 2004). However, the agarase used in the industry must exhibit high activity and stability at a temperature higher than the agar gelling temperature (1.5% of agar solution solidifies at 32–43 °C). In the present study, rAgaB-4 exhibited high activity and stability at 45 °C. Therefore, it can be used to produce neoagarotetraose.

Studies have shown that neoagarotetraose exhibits various biological activities, and because of its nontoxic properties, neoagarotetraose can be used in cosmetic, health food, and pharmaceutical industries (Jang et al. 2009; Hong et al. 2017b). For example, Jang et al. (2009) pointed out that 0.1 μg/mL neoagarotetraose could reduce the melanin content in murine melanoma B16F10 cells and could inhibit tyrosinase activity in B16F10 cells and mushroom tyrosinase activity in vitro; thus, it is a potential skin-whitening agent. Zhang et al. (2017) reported that neoagarotetraose could protect mice from intense exercise-induced fatigue damage by regulating the composition and function of intestinal microbes, and that it could be used as an ingredient in health foods. Wang et al. (2017) demonstrated that neoagarotetraose is a potential anti-inflammatory agent because it inhibits anti-inflammatory reactions in LPS-induced macrophages. In the future, we will use rAgaB-4 to hydrolyze agar and further investigate whether neoagarotetraose exhibits other biological active properties, such as anticancer and immunomodulatory activities.

The present study focused on AgaB-4 from *P. agarexedens* BCRC 17346. The results showed that rAgaB-4 exhibits β-agarase activity and can be used for the recovery of DNA from agarose gel and for the production of neoagarotetraose. Future research should focus on the cloning and expression of *agaB*-*1*, *agaB*-*2*, and *agaB*-*3* from *P. agarexedens* BCRC 17346 and should also evaluate whether recombinant proteins exhibit agarase activity.

## Additional file

**Additional file 1: Figure S1.** Multiple amino acid sequence alignment of AgaB-4 with known β-agarases from the GH50 family, including AgaW from *Cohnella* sp. LGH, Aga50D from *Saccharophagus degradans* 2-40, AgWH50A from *Agarivorans gilvus* WH0801, and HZ2 from *Agarivorans* sp. HZ105. A partially conserved catalytic residue of GH50 family is underlined in blue. *Filled triangles* indicate the active sites of Aga50D. **Figure S2.** SDS-PAGE analysis of total cell lysates (T), soluble (S), and insoluble (I) protein fractions from *E. coli* BL21 (DE3)(pET-AgaB-4) expressing rAgaB-4 after induction for 4 and 24 h at **a** 37 °C, **b** 30 °C, **c** 25 °C, **d** 20 °C, and **e** 16 °C with 0.1 mM IPTG added to the culture. *Lane M*, PageRuler™ Prestained Protein Ladder. The arrow indicates the protein bands of rAgaB-4. **Figure S3.** Recovery of pUC19 from low-melting point agarose by rAgaB-4. *Lane M*, 1-kb DNA Ladder; *lane 1*, original pUC19; *lane 2*, recovered pUC19 from low-melting point agarose.

## Abbreviations

rAgaB-4: recombinant AgaB-4
GH: glycoside hydrolase
BCRC: Bioresource Collection and Research Center
LB: Luria–Bertani
PCR: polymerase chain reaction
BLAST: basic local alignment search tool
OD_600_: optical density at 600 nm
IPTG: isopropyl-β-d-thiogalactopyranoside
SDS-PAGE: sodium dodecyl sulfate polyacrylamide gel electrophoresis
LMP: low-melting point
DNS: 3,5-Dinitrosalicylic acid
EDTA: ethylenediaminetetraacetic acid
HMP: high-melting point
TLC: thin layer chromatography
CAZy: carbohydrate-active enzymes
