# Supplementary material for: Molecular cloning, expression, and functional characterization of the β-agarase AgaB-4 from Paenibacillus agarexedens
Source: AMB Express. 2018 Mar 28;8:49. doi: 10.1186/s13568-018-0581-8 (PMC5874223; doi:10.1186/s13568-018-0581-8)
Supplement: Supplementary file 1 — Additional file 1: Figure S1. Multiple amino acid sequence alignment of AgaB-4 with known β-agarases from the GH50 family, including AgaW from Cohnella sp. LGH, Aga50D from Saccharophagus degradans 2-40, AgWH50A from Agarivorans gilvus WH0801, and HZ2 from Agarivorans sp. HZ105. A partially conserved catalytic residue of GH50 family is underlined in blue. Filled triangles indicate the active sites of Aga50D. Figure S2. SDS-PAGE analysis of total cell lysates (T), soluble (S), and insoluble (I) protein fractions from E. coli BL21 (DE3)(pET-AgaB-4) expressing rAgaB-4 after induction for 4 and 24 h at a 37 °C, b 30 °C, c 25 °C, d 20 °C, and e 16 °C with 0.1 mM IPTG added to the culture. Lane M, PageRuler™ Prestained Protein Ladder. The arrow indicates the protein bands of rAgaB-4. Figure S3. Recovery of pUC19 from low-melting point agarose by rAgaB-4. Lane M, 1-kb DNA Ladder; lane 1, original pUC19; lane 2, recovered pUC19 from low-melting point agarose. [file 13568_2018_581_MOESM1_ESM.doc]

**Supplementary materials**

**AMB Express**

**Molecular cloning, expression, and functional characterization of the β-agarase AgaB-4 from *Paenibacillus agarexedens***

**Zeng-Weng Chen^1^ · Hui-Jie Lin^1^ · Wen-Cheng Huang^1^ · Shih-Ling Hsuan^2^ · Jiunn-Horng Lin^1^ · Jyh-Perng Wang^1^***

^1^Animal Technology Laboratories, Agricultural Technology Research Institute, No.52, Kedong 2nd Rd., Zhunan Township, Miaoli County 350, Taiwan, ROC

^2^Graduate Institute of Veterinary Pathobiology, National Chung Hsing University, 250 Kuo Kuang Road, Taichung, 402,Taiwan, ROC

Correspondence: Jyh-Perng Wang

E-mail: [jpwang@mail.atri.org.tw](mailto:jpwang@mail.atri.org.tw)

Phone: +(886)-37-585889

Fax: +(886)-37-585850

**Supplementary Figures**

**Fig. S1** Multiple amino acid sequence alignment of AgaB-4 with known β-agarases from the GH50 family, including AgaW from *Cohnella* sp. LGH, Aga50D from *Saccharophagus degradans* 2-40, AgWH50A from *Agarivorans gilvus* WH0801, and HZ2 from *Agarivorans* sp. HZ105. A partially conserved catalytic residue of GH50 family is underlined in blue. *Filled triangles* indicate the active sites of Aga50D.

**
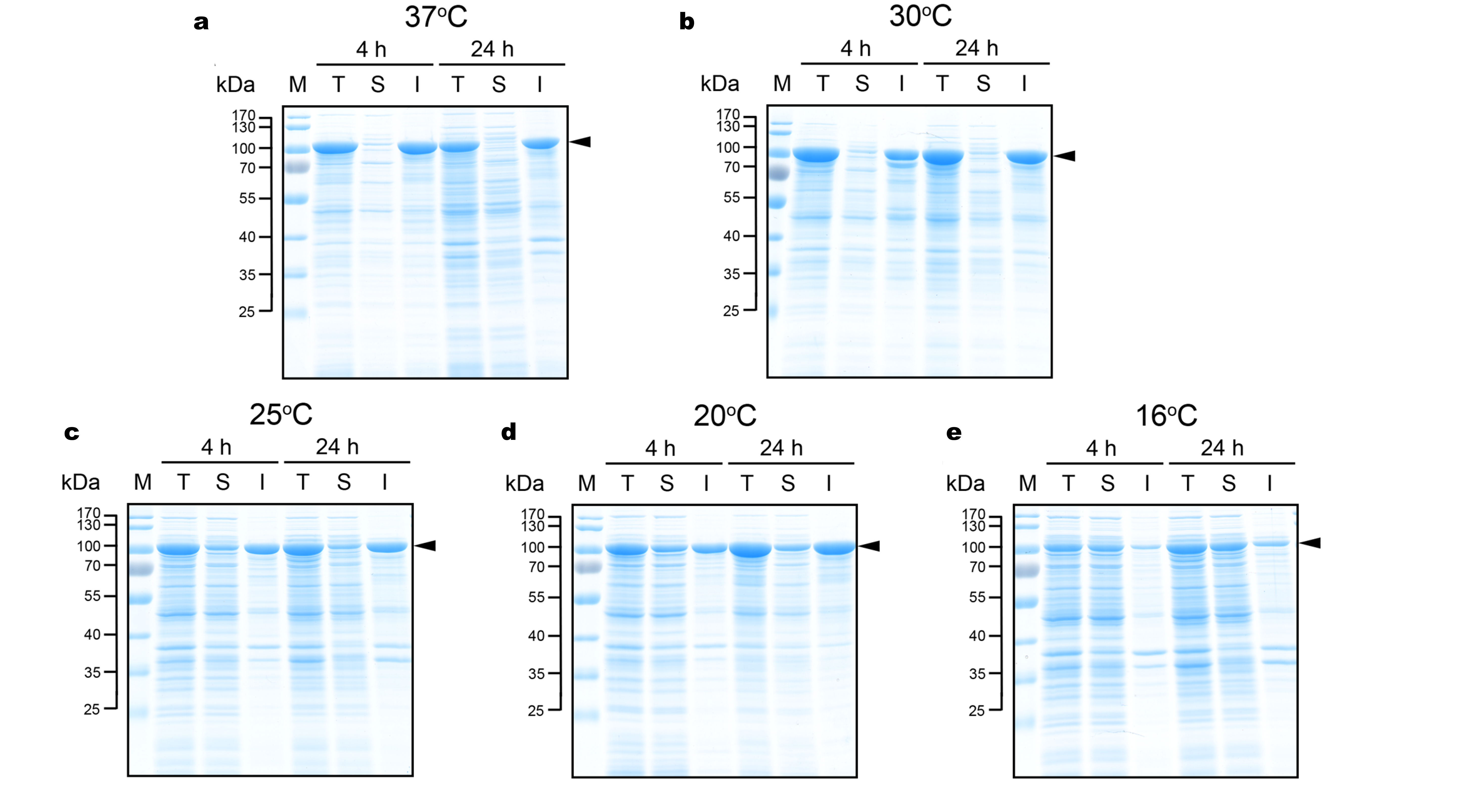
**

**Fig. S2** SDS-PAGE analysis of total cell lysates (T), soluble (S), and insoluble (I) protein fractions from *E. coli* BL21 (DE3)(pET-AgaB-4) expressing rAgaB-4 after induction for 4 and 24 h at **a** 37°C, **b** 30°C, **c** 25°C, **d** 20°C, and **e** 16°C with 0.1 mM IPTG added to the culture. *Lane M*, PageRuler^TM^ Prestained Protein Ladder. The arrow indicates the protein bands of rAgaB-4.

**Fig. S3** Recovery of pUC19 from low-melting point agarose by rAgaB-4. *Lane M*, 1-kb DNA Ladder; *lane 1*, original pUC19; *lane 2*, recovered pUC19 from low-melting point agarose.
